# The features and qualities of online training modules in research ethics: a case study evaluating their institutional application for the University of Botswana

**DOI:** 10.1080/11287462.2019.1592305

**Published:** 2019-03-26

**Authors:** Dolly Mogomotsi Ntseane, Joseph Ali, Kristina Hallez, Boikanyo Mokgweetsi, Mary Kasule, Nancy E. Kass

**Affiliations:** a Department of Social Work, University of Botswana, Gaborone, Botswana; b Johns Hopkins Berman Institute of Bioethics, Baltimore, MD, USA; c Johns Hopkins Bloomberg School of Public Health, Baltimore, MD, USA; d Department of Statistics, University of Botswana, Gaborone, Botswana; e Office of Research and Development, University of Botswana, Gaborone, Botswana

**Keywords:** Botswana, online research ethics courses, capacity development, ethical review

## Abstract

Research ethics remains a cornerstone of the scientific enterprise as it defines the boundaries of responsible conduct of research. Our aim was to systematically identify, review and test online training courses in research ethics which could be considered most appropriate for future training at the University of Botswana (UB). We used an evaluative tool that included both descriptive and evaluative criteria for assessing the strengths, weaknesses and appropriateness of 10 online research ethics courses which are publicly accessible. We then assembled Focus Group Discussions (FGDs) to engage the UB community to select the best 2–3 online courses that are considered most suited for use in future training of research ethics at UB. Twenty respondents participated in three FGDs. Our findings show that there is limited research ethics training capacity in low resourced academic institutions like UB. Online training opportunities could be used to address this challenge. Our analysis reveal that out of the 10 online courses reviewed, CITI program, Family Health International, and Training and Resource in Research Ethics Evaluation have characteristics that would make them suitable for utilization in Botswana. We believe the findings from this case study will be of value to other similarly situated research institutions.

## Introduction

Research ethics remains a cornerstone of the scientific enterprise as it defines the boundaries of responsible conduct of research and contributes to better management and governance of research. It also contributes to sustainability of further research and public trust in research. Opportunities for research ethics training for individuals in low- and middle-income countries (LMICs) has increased over the last two decades (Hyder, Harrison, Kass, & Maman, 2007; Hyder et al., 2013; Kass et al., 2007; Ndebele et al., 2014; Silverman, Strosberg, Luna, Philpott, & Hemmerle, 2013). This growth has come about, in part, as a response to demand for greater attention to the needs and concerns of human subjects and research populations participating in behavioral and clinical research in LMICs (Millum, Grady, Keusch, & Sina, 2013). With the growing necessity to build research ethics capacity, Fogarty International Center (FIC) launched the International Bioethics Education and Career Award in 2000 to provide grants for health professionals and academics from these countries to undergo research ethics training (Hyder et al., 2007; Millum, Sina, & Glass, 2015). Ali et al. (2014) notes that from 2000–2012, NIH-FIC awarded over US $40 million in research ethics capacity development for LMICs with nearly half of the grants supporting training in sub-Saharan Africa. Available data shows that since the launch of this training, remarkable achievements have been made. According to Matar, Garner, Millum, Sina, and Silverman (2014, p. 12), “since 2000, 74 countries have implemented 20 funded programs with approximately 600 masters level trainees graduating from these programs”. Further, Ndebele and colleagues report that since 2001, 275 African professionals have received long-term research ethics training. In addition, 19 countries in the region have created research ethics curriculum (Ndebele et al., 2014). A recent study evaluating the impact of Fogarty bioethics training programs (FBTPs) revealed that former trainees are now making significant contribution in their home countries in the areas of teaching, research and as members of Institutional Review Boards (IRBs) (FABTP, 2016; Kass, Ali, Hallez, & Hyder, 2016).

Consistent with this progress, institutions of higher learning in sub-Saharan region seem to be requiring increased training capacity for researchers (Ajuwon & Kass, 2008; Ateudjieu et al., 2010; Coetzee, Hoffmann, & de Roubaix, 2015; Hyder et al., 2013; Millum et al., 2013). Recent studies conducted in Botswana, however, show that limited research ethics training is available for UB academic staff, Institutional Review Board (IRB) members and students (Barchi, Kasimatis-Singleton, Kasule, Khulumani, & Merz, 2013; Barchi, Matlagela, Jones, Kebaabetswe, & Merz, 2015; Hyder et al., 2013; Ndebele et al., 2014; Ralefala, Ali, Kass, & Hyder, 2016). Training that has taken place has been in the form of short workshops and seminars for academic staff and IRB members (Barchi et al., 2013, 2015; Ralefala et al., 2016). A few academic staff members teaching research methods courses have also made efforts to integrate research ethics topics in their courses (Hyder et al., 2013).

Due to resource challenges, development of face-to-face institutionalized training programs has been difficult, and online platforms are increasingly becoming popular in LMICs since they are cost effective, flexible and have the capacity to reach a large audiences (Aggarwal et al., 2011; Caravias, 2014; Curran, Fleet, & Kirby, 2010; Gedera, 2014; Marrinan, Firth, Hipgrave, & Jimenez-Soto, 2015; Silverman et al., 2013; Stacey & Wiesenberg, 2007; Williams et al., 2014). However, online courses are criticized for being brief, compliance oriented and requiring stable internet connectivity (Allen & Seaman, 2011; Caravias, 2014; Ogunrin, Ogundiran, & Adebamowo, 2013).

In the literature, there are very few studies where online courses in research ethics were adapted through the utilization of community participation approach. Included amongst these are those which were conducted in Nigeria and amongst American Indian and Alaskan communities (Ogunrin et al., 2013; Pearson, Parker, Fisher, & Moreno, 2014). In both studies, the Collaborative Institutional Training Initiative (CITI) course was adapted using a randomized design to develop online training courses that are culturally sensitive and relevant to the needs of research ethics committees and researchers in these communities.

This case study sought to collect, document and systematically evaluate the features and qualities of several publically available introductory online training courses in research ethics to determine whether any may be suitable for use as a required training for researchers in the largest public university in Botswana. We believe the methods and findings from this case study will be of value to other similarly situated research institutions in lower resource settings.

## Methods

### Study setting

This study was carried out at the University of Botswana (UB). There are roughly 18,000 students enrolled at undergraduate, masters and PhD programs and over 900 academic staff across eight faculties (Statistics Botswana, 2015). Like many institutions of higher learning in lower resource settings, UB has developed research ethics policies and structures to ensure that researchers adhere to the highest professional and ethical standards when conducting research. At the operational level, the Office of Research and Development (ORD) is responsible for policy formulation and administration of two IRBs: Bio-Medical and Socio-Behavioral. IRBs provide oversight of all human research conducted by staff and students as well as international scholars. Over the years the volume and complexity of research at UB has increased. For example, in 2013 over 800 proposals were submitted by undergraduate and postgraduate students as well as academic staff and collaborators (Office of Research & Development, 2018). Despite this increase, and unlike many other universities, research ethics training is not a mandatory requirement for researchers prior to submitting applications for ethics review. This study was intended to help address the existing training gap by better understanding training options available.

## Study design

This case study utilized a cross sectional evaluative design to assess a range of publicly accessible introductory, self-administered research ethics courses that include modules on the designated topics. The study employed a mixed research approach; qualitative, quantitative and desk review. Throughout data collection, the researchers engaged UB Expert Advisory Group (EAG) to get expert advice on research ethics topics that are best suited for UB and any other matters related to this research. The study was conducted between November 2016 and June 2017.

## Sampling

Eight (8) academic staff members were recruited using purposive sampling from the faculties of Social Science, Health Science, Medicine, Humanities and Education. Only academic members who are currently engaged in behavioral or clinical research were selected to participate. Further, 8 students from various faculties were also selected purposively through the assistance of Graduate Coordinators. Students who were likely to submit their research proposals to IRB (or who have already done so) were included in the selection process. Finally, 3 IRB members were purposively selected from the UB Biomedical IRB and 3 IRB members from the UB Behavioral committee.

## Study tool

An evaluation tool that included both descriptive and quality evaluative criteria was developed to assess the strengths, weaknesses and appropriateness of courses for training of UB academic staff, students and IRB members. To assess the descriptive features of the courses, a spread sheet was developed showing the specific features and nature of each online professional training course such as aim of the course, topics, layout, teaching methods, assessment, and cost (See Table 1). To create the quality evaluative tool, two comprehensive evaluation criteria matrix were identified from the literature and reviewed: an evaluation matrix based on recommended best practices in eLearning Silverman et al. (2013) and the Southern Regional Education Board (SREB) Checklist for Evaluating Online Courses (SREB, 2006). The quality assessment matrix included four sections for assessing the quality of each online course namely; section A: content, section B: layout and instructional design, section C: technology and section D: relevance to UB Context***.*** Finally, a rating scale was used to evaluate the courses: 3 points = Meets criteria, 2 points = Partially meets criteria, 1 point = Does not meet criteria.

**Table 1.** Characteristics of Identified Online Research Ethics Courses.

Online Courses	Goal	Layout/Design	Topics/Content	Teaching Methods	Assessment	Duration	Cost	Admission	Context/Relevance to UB
CITI (HSR – Biomedical)	Introduction to biomedical research with a focus on the protection of human subjects.	16 broad topics and sub-topics	Belmont Report and CITI History of Ethics of Human Subjects Research Basic Institutional Review Board (IRB) Regulations and –Review Process -Informed Consent -Social and Behavioral Research (SBR) for Biomedical Researchers Records-Based Research Genetic Research in Human Populations Recognizing and reporting unanticipated problems involving risks to subjects or others in Biomedical and or Protections Vulnerable Subjects – Research Involving Prisoners Vulnerable Subjects – Research Involving Children Vulnerable Subjects – Research Involving Pregnant Women, Human Fetuses and Neonates Avoiding Group Harm – US Research Perspectives Avoiding Group Harm – International Research Perspectives FDA –Regulated Research Research and HIPAA Privacy Protections	Online course using case studies, video, slide show presentation. This could complement an in-person training experience.	Students must pass each module quiz with 70% or above Award of Certificate upon completion	Three hours (3 h)	USD $3500/year paid by the institution for all the users or $110/course for independent learner	Undergraduate, graduate students to postdoctoral researchers, administrators and IRB members	Yes, topics are relevant to UB; in particular health scientists
CITI Program – HSR – Socio-behavioral-education	Introduction to social-behavioral-educational research with a focus on the protection of human subjects.	13 broad topics and sub-topics	Belmont Report and CITI Course Introduction History and Ethical Principles Defining Research with Human Subjects The Federal Regulations Assessing Risk Informed Consent Privacy and Confidentiality Research with Prisoners Research with Children Research in Public Elementary and Secondary Schools International Research Internet Based Research Unanticipated Problems and Reporting Requirements in Social and Behavioral Research	Online course using case studies, video, slide presentation. This could complement an in-person training experience.	Students must pass each module quiz with 70% or above Award of certificate upon completion of the course	Three hours (3 h)	USD $3500/year paid by the institution for all the users or $110/course for independent learner	Undergraduate, graduate students to postdoctoral researchers, administrators and IRB members	Yes, topics are relevant to UB; in particular, teachers and social scientists
Fhi360	Basic and accessible level of training appropriate for individuals from different professional backgrounds and world regions.	8 broad topics and sub-topics	The principles of research ethics The development of contemporary research ethics Informed consent Responsibilities of Research Ethics Committees Responsibilities of Sponsors and Researchers Community participation in the research process	Color slides for online presentation, case study analysis and discussion points, option for facilitator; if need be; additional resources and useful internet sites – easy to navigate through the course	Evaluation includes a post-test and a curriculum evaluation form. Award of certificate at the completion of the course	Not stated	FREE	Open to all researchers, IRB members and public	Yes the topics are relevant to UB; In particular – health scientists
TREEE	Basics of research ethics evaluation and the broader context of research ethics.	6 broad topics sub-topics	Historical Overview Core values and concepts of Ethics for Research Involving Humans Overview of normative frameworks applicable to health research involving humans National Instruments Institutional Requirements Introduction to Research ethics evaluation	Online lectures, case studies, text, videos, resources are available for students (also includes country specific national guidelines and IRB documents).	Students must get 70% or above on the self-assessment test Award of certificate	Not stated	FREE	Open to all who participate in research including IRB members, research teams in the social science and health sciences as well as political authorities etc.	Yes, topics are relevant to UB. About 148 participants from Botswana have been awarded certificates.
CITI Program (RCR)	A foundation for institutions that are working to promote Responsible Conduct of Research (RCR)	12 broad topics and sub-topics	Introduction to RCR Authorship Collaborative Research Conflicts of Interest Data Management Financial Responsibility Mentoring Peer Review Plagiarism Research Involving Human Subjects Research Misconduct Using Animal Subjects in Research	Online lectures, case studies, video, slide presentation. The CITI could complement an in-person training experience.	Students must pass each module quiz with 70% or above Award of certificate	Three hours (3 h)	USD $3500/year paid by the institution for all the users or $110/course for independent learner	Undergraduates, graduates and postdoctoral researchers, administrators, IRB members and other researchers	No. the course is focused more on responsible conduct of research.
Ethics of Human Subject Research- JHSPH	Ethics concepts as they apply to questions and challenges in conducting human subject research. The aim is to increase students’ knowledge and skills to recognize and consider ethical issues that arise in the conduct of human subject research.	12 broad topics and sub-topics	History of Research Ethics in US and South Asia Ethics Principles Ethics Framework Informed Consent: What it is and what it includes Informed Consent: What contributes to a Good Process Risk/Benefits Assessment Honesty in Science: Integrity and Authorship and Conflict of Interest Privacy/Confidentiality Justice: Applied	Online Lectures, Case studies, MP3 enhanced. Other useful resources are available for students	Not stated Information is not available on the award of certificate	Not stated	FREE	Not stated	No. Primarily focused on US and India case examples
University of Maryland	Ethical and regulatory aspects of human subjects research and skills needed to analyze ethical issues that arise in the conduct and coordination of research in domestic and international arenas	6 broad topics and sub-topics	Introduction ton Research Ethics -3 credits Issues in International Research Ethics – 3 credits Introduction to Ethical Theory – 2 credits Institutional Review Board – 2 credits Responsible Conduct of Research in International Affairs – 1 credit Ethics of Globalization – 1 credit	Online Lectures, Course work, Case analysis Other useful resources are available for students	Course work assignments Award of certificate.	One year, participants can start the program at the start of any term. Participants are given up to 3 years to complete	US$ 766 cost per credit hour for out of State learners	Undergraduates, graduates and postdoctoral researchers, administrators, IRB members and other researchers	Yes, however the course is only suitable for participants who can afford to travel to Maryland. This may not be feasible for majority of researchers
JHSPH Open Courseware	Ethics of human subjects research	8 broad topics and sub-topics	Ethical theory and principles History of research ethics Contemporary issues in research ethics Balancing the benefits and harms of participating in research Ethical issues in study design Informed Institutional Review Board Selection of research participants Ethical issues in vaccine research	Lectures, moderated discussions, case study analysis. Lecture materials, readings are available for students.	Participation in moderated discussions, informed consent exercise and written case analysis Award of certificate.	The course takes 8 weeks to complete	Not stated	Not stated	Yes, however, the course takes 8 weeks to complete which may not be feasible for majority of researchers
Global Health Training Centre	Not stated	14 broad topics and sub-topics	Introduction to research ethics Principles and Guidelines Evaluating risks and benefits Issues of study design Understanding vulnerability Obligations of research participants Engaging with the community Privacy and confidentiality Informed consent Other ethical issues Bio banks and genomic research Including people with disability Women in biomedical research Research in public health emergencies	Online course topics, case studies, videos. Resource library available and other resources	An inbuilt quiz to test knowledge. A minimum of 80% is required as pass mark Award of certificate.	Not stated	FREE	Course is designed and produced for WHO internal staff; but made available for all interested	Yes the topics are relevant to UB. Researchers in the health settings would benefit more from this course.
University of Montana	Foundation for institutions that are working to promote responsible conduct for research.	6 broad topics and sub-topics	Ethical issues in research – A framework Interpersonal Responsibility Institutional Responsibility Professional Responsibility Animals in Research Human Participation in research	Course topics, case studies. Resource library available	An inbuilt quiz to test knowledge Award of certificate	Not stated	FREE	Course offered to graduate students and investigators	A few topics are relevant to UB.

Key: Online Courses: Online courses reviewed (Introduction to Research Ethics); Goal: The overall goal of the course; Layout and Design – how the course is structured; Topics/Content: Topics covered; Teaching methods: Different types of teaching methods used; Assessment – How the course is assessed; Duration: Duration of the course; Cost – Fees charged; Admission: Admission requirements; Context – Relevance to Botswana context.

## Data collection procedures

To systematically collect online and publicly accessible introductory self-administered research ethics courses, an extensive web and literature search was conducted. Key search terms were applied including: research ethics &training & online; research ethics & training & module; online research ethics & curriculum; online training & Fogarty; capacity building & online research ethics. In addition, outreach was also made to key ethics experts via email to provide information on available online courses. These included National Institutes of Health, Fogarty International Center, Johns Hopkins University research ethics faculty, and the alumni network of the Johns Hopkins Fogarty African Bioethics Training Program, Research Ethics Committee Association of Southern Africa, as well as other NIH Fogarty research ethics training program grantees and corresponding authors on relevant manuscripts.

To collect information from informants on what they saw as the relative strengths and weaknesses of the research ethics courses, Focus Group Discussions (FGDs) were conducted with academic staff, graduate students and IRB members from UB. Using a semi structured interview guide, participants were asked to identify strengths and weaknesses of the 2–3 selected courses, which one they liked and disliked as well as the one they considered best suited for UB and why? A few days before this engagement, the target audience was sent a document via email detailing 10 online courses to browse through and evaluate (See Appendix). This process was followed by a focus group looking at a smaller number of candidate courses that received the highest scores from all the assessment criteria.

## Statistical analysis

Using Microsoft Excel, the analysis utilized the quantitative nature of ordinal variables through assigning numerical scores to categories of each criterion. Each course was assessed to determine if it met the set standard using the rating scale stated above. The total number of criteria was 31 with evaluation matrix of 310 [31×10] from all ten courses under consideration. Also, these criteria were subdivided into four sections as described in the study tools above. For each section and all sections combined, the frequencies and percentages of scores were obtained based on whether the course assessed met, partially met or did not meet the criteria. The descriptive statistics for courses grouped were carried out to determine the percentages on rating scale by sections. Course ranking was based on the total scores accumulated from all the sections.

## Qualitative analysis

Responses from FGDs were transcribed. Data were then coded sentence by sentence. Common themes, patterns and clusters were identified and organized systematically (Braun & Clarke, 2006; Huberman & Miles, 1994). Data were then grouped together into themes and sub-themes.

## Results

This section presents the findings of the study. Altogether, ten (10) online courses were identified and selected through web and literature search. This selection was also based on the topics that were identified by EAG as of high priority to be taught for research ethics training at UB namely; history of research ethics, ethical theory and principles, informed consent, vulnerable groups, genomic research, study design, community engagement, IRB process, regulations, and legal issues. Table 1 presents characteristics of the courses namely; course goal, layout, topics, teaching methods, assessment, duration, cost, admission criteria and relevance to UB. Table 2 on the other hand shows four quality assessment areas on each course; section A: content, section B: layout, section C: technology and section D: relevance to UB context.

**Table 2.** Quality assessment matrix.

Criterion	Section A: Content
CRITERIA_1	A clear, complete course overview and syllabus is provided
CRITERIA_2	The course goals and objectives are clearly defined, measurable and aligned and state what the participants will know or be able to do at the end of the course
CRITERIA_3	The course content and assignments are of sufficient rigor, depth and breadth to teach the standards being addressed
CRITERIA_4	Information literacy and communication skills are incorporated and taught as an integral part of the curriculum
CRITERIA_5	Information concerning academic integrity are clearly stated
CRITERIA_6	Assessment and evaluation use multiple methods
CRITERIA_7	Learners have opportunity to communicate feedback regarding the course structure and content
CRITERIA_8	The grading policy and practices are clearly stated and easy to understand
CRITERIA_9	Learners are provided with a certificate upon successful completion of the course
	Section B: Layout and instructional design
CRITERIA_1	The course is organized into units and lessons
CRITERIA_2	The course unit overview describes the objectives, activities and resources that frame the unit
CRITERIA_3	Each lesson includes a lesson overview, content and activities and provides multiple learning opportunities for students to master the content
CRITERIA_4	Navigation cues are present, clearly identifiable
CRITERIA_5	The format is uncluttered making it easy for learners to understand the content
CRITERIA_6	Images and graphics are clear and optimized for efficient loading
CRITERIA_7	The content is broken into small, incremental learning steps and organizes in a logical, consistent sequence
CRITERIA_8	Case studies are used effectively in the text
CRITERIA_9	Students have access to resources that enrich the course content
	SectionC:Technology
CRITERIA_1	The course architecture permits the online teacher to add content, activities and assessment to extend learning opportunities
CRITERIA_2	The course is easy to navigate
CRITERIA_3	Hardware, Web browser and software requirements are specified
CRITERIA_4	The course provider offers the course learner with technical assistance to manage the course
CRITERIA_5	The course uses video to reinforce learning
	SectionD:Relevancetouniversity of Botswana context
CRITERIA_1	The course is relevant to Botswana cultural context and educational set-up
CRITERIA_2	The course topics meet the needs of UB and Botswana research landscape
CRITERIA_3	The course is flexible to allow local materials to be added
CRITERIA_4	Blended approach could be infused into the course
CRITERIA_5	The course duration is reasonable
CRITERIA_6	The course is suitable for students and academic staff
CRITERIA_7	The course is free

**Figure 1.**
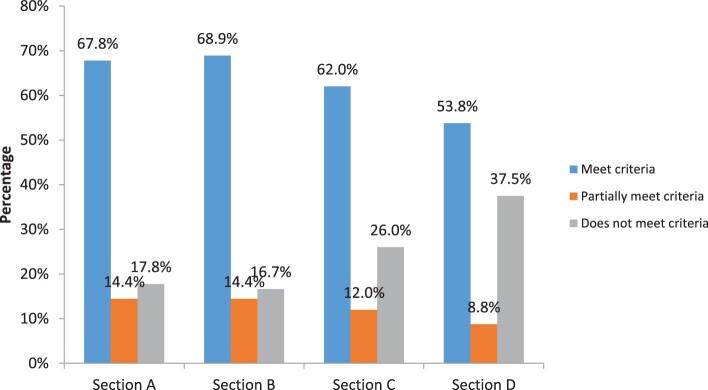
Percentage of criteria receiving a score of meeting criteria, partially meet criteria and does not meet criteria for all online courses classified by sections. Key: Section A: Content, Section B: Layout and Instructional Design, Section C: Technology, Section D: Relevance to UB context.

In Figure 1, we show the aggregate scores distribution for the criteria by sections. As revealed in Figure 1, the highest score of meet criteria was observed in section B with 69% followed by section A with 68%. The lowest score was recorded in section D with 54%. For the score of not meet criteria, section D dominates with 38%. Generally, this indicates that the criteria in section D received low scores compared to that of other sections.

**Figure 2.**
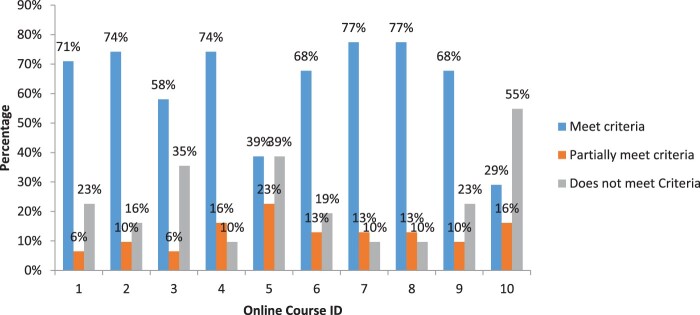
Percentage of criteria receiving rating score for each of the selected online ethics course. Key course ID and ethics course name: 1, Introduction to the ethics of human subject research; 2, Training and Resource in Research Ethics Evaluation (TREEE); 3, JHSPH Open Courseware -Ethics of Human Subjects Research; 4, Family Health International (fhi360); 5, Global Health Training Centre – Research Ethics Online Training; 6, CITI Program – Responsible Conduct of Research (RCR); 7, CITI Program-Human Subjects Research (HSR – Biomedical); 8, CITI Program – Human Subjects Research (HSR – Socio-behavioral-education); 9, University of Maryland-Online Research Ethics Certificate; 10, University of Montana with Office of Research Integrity.

Further, Figure 2 presents results showing the percentage of the 30 criteria receiving scores of “meets,” “partially meets” and “does not meet” criteria for each course. The CITI (HSR – Biomedical) and CITI (HSR – Socio-behavioral-education) modules most frequently received the highest score of “meets” criteria with 77%, followed by TREEE and FHI with 74% each. For CITI (HSR – Biomedical) and CITI (HSR – Socio-behavioral-education) modules the highest scores of meet criteria were observed in section C (80%), followed by section A and B with 78% each and lastly section D with 75%. As for TREEE, the highest score of meet criteria was observed in section D (88%) and the lowest was in section C (60%). The FHI module recorded the highest score of meet criteria in section A and B (with 78% each) and lowest score was in section C (60%). The course that received the least score of “meets” criteria was University of Montana (UM) with 29%. Lastly, three courses (University of Montana with Office of Research Integrity, Global Health Training Centre – Research Ethics Online Training and JHSPH Open Courseware – Ethics of Human Subjects Research) received at least 35% for the score of “does not meet” criteria

Table 3 on the other hand presents the overall scorings for each course's relevance to UB. For rating of meeting criteria, TREEE received the highest score of 88% followed by CITI and FHI with 75% each; and UM attained the least score of 25%.

**Table 3.** Relevance to University of Botswana context: Overall scores.

Course	Meets criteria	Partially meets criteria	Does not meet criteria	Total
Training and Resource in Research Ethics Evaluation (TREEE)	7 (87.5%)	0 (0.0%)	1 (12.5%)	8 (100.0%)
CITI Program-Human Subjects Research (HSR – Biomedical)	6 (75.0%)	1 (12.5%)	1 (12.5%)	8 (100.0%)
CITI Program – Human Subjects Research (HSR – Socio-behavioral-education)	6 (75.0%)	1 (12.5%)	1 (12.5%)	8 (100.0%)
Family Health International (fhi360)	6 (75.0%)	1 (12.5%)	1 (12.5%)	8 (100.0%)
Introduction to the ethics of human subject research	4 (50.0%)	0 (0.0%)	4 (50.0%)	8 (100.0%)
Global Health Training Centre – Research Ethics Online Training	3 (37.5%)	2 (25.0%)	3 (37.5%)	8 (100.0%)
CITI Program – Responsible Conduct of Research (RCR)	3 (37.5%)	1 (12.5%)	4 (50.0%)	8 (100.0%)
University of Maryland-Online Research Ethics Certificate	3 (37.5%)	1 (12.5%)	4 (50.0%)	8 (100.0%)
JHSPH Open Courseware -Ethics of Human Subjects Research	3 (37.5%)	0 (0.0%)	5 (62.5%)	8 (100.0%)
University of Montana with Office of Research Integrity	2 (25.0%)	0 (0.0%)	6 (75.0%)	8 (100.0%)

Finally, the total scores and ranking of the selected online courses based on the total scoring from all the assessment criteria are shown in Table 4. The CITI – Human Subjects Research (HSR – Biomedical) module and CITI Human Subjects Research (HSR – Socio-behavioral-education) module were ranked number one with total score of 89% respectively, followed by the Family Health International (fhi360) with score of 88% and the Training and Resource in Research Ethics Evaluation (TREEE) that was ranked third with a score of 86%.

**Table 4.** Ranking of online ethics courses based on the total scores received from all the assessment criteria.

Course	Scores				Total	Rank
	Section A	Section B	Section C	Section D		
CITI Program-Human Subjects Research (HSR – Biomedical)	24 (88.9%)	24 (88.9%)	14 (93.3%)	21 (87.5%)	83 (89.2%)	1
CITI Program – Human Subjects Research (HSR – Socio-behavioural-education)	24 (88.9%)	24 (88.9%)	14 (93.3%)	21 (87.5%)	83 (89.2%)	1
Family Health International (fhi360)	25 (92.6%)	25 (92.6%)	11 (73.3%)	21 (87.5%)	82 (88.2%)	3
Training and Resource in Research Ethics Evaluation (TREEE)	23 (85.2%)	23 (85.2%)	12 (80.0%)	22 (91.7%)	80 (86.0%)	4
CITI Program – Responsible Conduct of Research (RCR)	24 (88.9%)	24 (88.9%)	14 (93.3%)	15 (62.5%)	77 (82.8%)	5
Introduction to the ethics of human subject research	23 (85.2%)	24 (88.9%)	14 (93.3%)	16 (66.7%)	77 (82.8%)	5
University of Maryland-Online Research Ethics Certificate	26 (96.3%)	24 (88.9%)	11 (73.3%)	15 (62.5%)	76 (81.7%)	7
JHSPH Open Courseware -Ethics of Human Subjects Research	24 (88.9%)	21 (77.8%)	10 (66.7%)	14 (58.3%)	69 (74.2%)	8
Global Health Training Centre – Research Ethics Online Training	15 (55.6%)	20 (74.1%)	11 (73.3%)	16 (66.7%)	62 (66.7%)	9
University of Montana with Office of Research Integrity	17 (63.0%)	18 (66.7%)	7 (46.7%)	12 (50.0%)	54 (58.1%)	10

## UB community deliberations

Participants in the three FGDs deliberated on what they considered to be the strengths and weaknesses of the 2–3 best selected online courses. Below we give a brief presentation of the discussions.

## CITI program (HSR – Biomedical and HSR – Socio-behavioral-education)

Commenting on the strengths of the CITI program, majority of participants seemed to agree that it is more detailed and comprehensive compared to other programs considering that it offers introductory courses to both biomedical and socio-behavioral education research. Many participants were of the view that topics under HSR-Biomedical module such as IRB review process, informed consent, and records based research and genetics research in human populations are very critical for the UB Bio-Medical IRB. In addition, the module on socio-behavioral-education could assist researchers in the social sciences, humanities and education. Some observed that currently many researchers at UB conduct studies in primary and secondary schools; therefore there is need for capacity building on research with children in the school setting. As one participant explained,

the module on socio-behavioral-education will assist researchers in the social sciences, humanities and education. We receive a lot of proposals from researchers who are conducting studies in primary and secondary schools. This is an area which we need to strengthen … we often just look at assent issues and that's all when we consider research with children; but there is more to it than just these issues.

Another noted

I had the benefit of doing both courses in one of our workshops here at UB, and other online courses. Besides the cost, CITI is the one we could pick. If researchers are aware that UB is paying, they might feel its importance.

Further, participants also highlighted the fact that the CITI program offers other courses that researchers could take in addition to the introduction to research ethics such as Responsible Conduct in Research and Introduction to the Ethics of Human Subject Research. These would build additional knowledge which is much needed by IRB members and a wider audience at UB namely, undergraduate, graduate students, postdoctoral researchers and administrators.

Whilst an overwhelming majority of participants voted for CITI, some weaknesses were noted which could hinder the program being readily adapted. First, UB will have to pay an annual subscription fee ($3,500 USD at the time of this study) to provide this course for staff and students. Most participants lamented that currently UB is reported to be experiencing financial crisis and because of this, UB management may not readily agree to finance this program particularly if there are free online courses that researchers could take. Yet another concern expressed by a few people was that the CITI HSR – Biomedical program does not include ethical issues in primary and secondary schools; this is only addressed in the socio-behavioral-education course.

### FHI (fhi360)

Participants were in agreement that FHI could be considered as the second best choice because similar to CITI, the course includes topics suitable to UB such as ethics principles, informed consent, contemporary research ethics, to mention but a few. However, they singled out two topics included in FHI which they felt would be of great value to UB namely; responsibilities of IRB committees and community participation. Participants noted that there is a general lack of understanding by some UB researchers about the role of the IRB; as a result, they often question the decisions of the board and criticize the review process. Turning to community participation, participants in the IRB FGD reported that engaging communities in research is increasingly becoming important not only in Botswana but worldwide. They were of the view that community engagement ensures that informed consent is culturally appropriate, that research is responsive to community needs and that research benefits are shared with the community. Other strengths identified in the FGD were that FHI is designed for an international audience and it is free. For example, the program provides case studies from Africa, Asia and other developing countries.

Some weaknesses were observed. FHI was not believed to be as comprehensive as CITI given the current needs of the two IRBs at UB. Second, it tends to have a strong bias on health issues; as one participant observed,

Researchers are always complaining that it seems we are imposing bio-medical content. I see FHI as being restrictive on health, whereas CITI is more comprehensive as other researchers from education and social sciences will find the content applicable. I don't think it is fair to impose bio-medical into behavioral scientists.

### TREEE

Whilst the academic staff and IRB members seem to have a strong preference for CITI or FHI, students participating in the FGD were unanimous that TREEE was most suitable for them. Although students were instructed to focus on the top two online courses, they were quick to point out that TREEE covers topics that are more relevant for all academic disciplines including humanities, social science and business. Majority were of the view that the topics offered were more attractive and appealing to them and hence they felt they could benefit more from this program. Commenting on the fees charged by CITI one participant observed, “here at UB, we are told there is no money, we fear that the cost for this program may be transferred to us if the course is made compulsory.”

Nearly all students voted for TREEE because they reported that compared to the other two online platforms, it was designed in such a way that UB can add local content; hence they considered it more adaptable to the cultural and contextual needs of Botswana. Participants also made an observation that the fact that 148 Batswana have taken this course already means that it has been tested and proved beneficial (http://elearning.treee). It is worth noting that although TREEE fell to number 3 in ranking based on the total scores received from all the assessment criteria, it scored the highest by 88% followed by CITI with 75% and FHI with 75% in terms of relevance to UB.

#### Final adaptation of online courses

Consensus from the three focus groups was that in the short term, UB must consider making it mandatory for researchers and students to take an introductory online course on research ethics. A significant majority of academic staff and IRB members indicated that there is a need for training in research ethics for both staff and student researchers. A concern was raised that some members in the IRB have not received adequate training on research ethics. On the other hand, students were unanimous that they lack basic knowledge on research ethics. They reported that the information they get from research courses is very limited.

Regarding which online course is most suitable for UB, academic staff and IRB members preferred the CITI program, followed by FHI with a very close margin; while a majority of students voted for TREEE. Academic staff and IRB members were of the view that whilst these online courses seem to be beneficial, in the future a plan must be put in place to develop on-site to be offered to all students doing research and those interested in research ethics. A suggestion was made that when UB adapts an online course, efforts must be made to explore ways to infuse the local context. For example, issues related to risk, benefits, and incentives should be contextualized. In addition, ethical standards related to conducting research with indigenous communities must be explored. In this regard, UB could benchmark with universities in South Africa, Australia and New Zealand who have developed such guidelines.

Further, some participants from the staff focus group pointed out that topics offered by the University of Maryland and the University of Montana such as the impact of globalization on research, ethical issues involved in international research, ethical questions regarding international collaboration in research, responsible authorship, professional responsibility, peer review and internationalization are also very important for UB considering the current advancements in research. Therefore UB could mount a course that blends the courses from these two universities to build knowledge capacity for those who have already taken the basic course in research ethics.

## Discussion

The goal of this study was to systematically collect various online introductory courses and through the application of criteria and with input of key stakeholders at UB to identify the most appropriate course(s) for the UB community. Our findings reveal that training in research ethics is highly needed for students, researchers and academic staff UB. This observation was also highlighted by previous scholars (Ali, Hyder, & Kass, 2012; Barchi et al., 2013, 2015; Hyder et al., 2013; Silverman et al., 2013). In a more recent study Ralefala and colleagues reported that “there is need for targeted training in research ethics and IRB processes for junior and mid-career researchers and refresher training for all researchers at regular intervals” (Ralefala et al., 2016, p. 10). These researchers recommended that UB “should consider developing research ethics courses at graduate level and mandatory training for researchers to help build confidence and capacity outside the IRB in identifying and addressing ethical issues” (Ralefala et al., 2016, p. 11). This finding can be generalized to other academic institutions in LMICs who are facing similar challenges (Kass et al., 2016; Loue, 2014; Matar et al., 2014; Millum et al., 2013: Mzayek & Resnik, 2010).

The vision of UB is to become a research intensive institute which is internationally recognized for the volume, quality and impact of its research (University of Botswana, 2008). For this vision to be realized there is need for the UB management to consider intensifying building research ethics capacity for students and academic staff. Research produced must meet minimum ethical standards for it to be of high quality and impact. The findings of this study reveal that in the short term, UB could take advantage of publicly accessible online training courses to fill the existing training gap. The advantage of online vs traditional onsite training is that learners can access the course from their own space and at their preferred time (Aggarwal et al., 2011; Caravias, 2014; Curran et al., 2010; Gedera, 2014; Stacey & Wiesenberg, 2007). Compared to the on-site, online is cost-effective (Aggarwal et al., 2011) and finally, learners using the online mode are more self-directed and independent (Ogunrin et al., 2013; Stacey & Wiesenberg, 2007). However, this platform requires effective internet connectivity and technical capacity of learners and instructors (Barchi et al., 2013; Caravias, 2014; Marrinan et al., 2015). This limitation was highlighted by students in the FGD. To address this challenge, blended teaching approach could be adopted to enhance student engagement (Barchi et al., 2015; Kalichman, 2005; Marrinan et al., 2015; Muller, Swanepoel, & De Beer, 2010). These scholars are of the view that online courses that require interaction with faculty and which require students to do case analysis are more effective in fostering ethical skills. Regarding which online course UB could adapt for future use, this study found that CITI was liked best for its breath of topics and because it will serve the needs of the two IRBs. FHI on the other hand was voted for its emphasis on community engagement. Finally, TREE was liked for its relevance to the local setting. Although CITI emerged the most preferred, both faculty and students were concerned about the cost implication given the fact that UB is currently going through financial difficulties. Given this practical consideration, in the short term, UB could make it a requirement for all researchers applying for IRB review to take either FHI or TREE. Since FHI is more health focused, it could be taken by researchers in the health field whereas TREE could be taken by researchers in education and social science fields. In the long term, ORD office should engage UB management to subscribe for CITI.

Whilst online research ethics courses could be utilized in the short term, the findings from this research also echo the sentiments expressed by previous research that higher academic institutions in low resourced countries must institutionalize research ethics training through the mounting of undergraduate as well as graduate courses (Hyder et al., 2013; Ralefala et al., 2016). In the case of UB, there is a crop of faculty members who have been trained in research ethics (Kass & Hyder, 2015; Ndebele et al., 2014; Oukem-Boyer & Bukusi, 2015). At the time of this study, 10 faculty members had gone through FBTP short-term and long term training; two had attended courses in South Africa and one candidate attended the Brocher Summer Academy in the Ethics of Global Population Health on the Ethical Design of Randomized Studies for Economic Development**.** Considering the rate at which the university population is growing and the increase in volume and complexity of research conducted, there is still great need for training in research ethics and UB has not built sufficient capacity to supply the demand.

Finally, to our knowledge, this is the first study to utilize community engagement approach in adapting an online course to an academic institution. Throughout the study, consultations were held with various key stakeholders from the design the development of the descriptive and evaluative criteria, identification of topics of highest priority at UB and finally selection of the best 2–3 courses that may be adapted.

## Limitations

Data for this study consisted of publicly accessible research ethics courses available online. Although an extensive web and literature search was conducted, it is possible that we were not able to capture all details pertaining to descriptive and evaluative criteria. This limitation is likely to affect the aggregate scores for individual courses. Further, CITI programs could not be easily accessed online as learners are required to pay first. Hence focus groups had to rely heavily on data captured through the descriptive criteria. Yet another limitation is that although community engagement approach added value in the selection of the best suited course for UB, this methodology was time consuming and laborious as the process required much back and forth communication to reach consensus with EAG and other key stakeholders. It was not always possible to get prompt feedback that was needed to move the study to the next levels. On the other hand, students from humanities, environmental studies and health sciences did not participate in FGDs although they had been invited. Further, only undergraduate students participated in the focus groups. Finally, the findings presented in this study are from a fairly small sample of people from UB; others may have had other views.

## Appendix

### Brief Description of Online Courses

**JHSPH Open Courseware – Introduction to the Ethics of Human Subjects Research**: http://ocw.jhsph.edu/index.cfm/go/viewCourse/IntroHSR/coursePage/index/

The course introduces students to ethics concepts as they apply to questions and challenges in conducting human subject research. The aim is to increase students’ knowledge and skills to recognize and consider ethical issues that arise in the conduct of human subject research. Lectures are divided into 12 topics. Major topic include; history of research ethics in US and South Asia, principles, ethics framework, informed consent, risk/benefits assessment, honesty in science, integrity and authorship; conflict of interest. Each topic has sub-topics. Slides for each topic are presented in PDF format and the recorded lectures are presented in MP3. Users will have to listen to the recordings to be able to understand and follow the lesson. The course could take 3–8 h to complete.

**Training and Resource in Research Ethics Evaluation (TREEE)** http://elearning.trree.org/course/view

This is an introductory module that presents the basics of research ethics evaluation and the broader context of research ethics. The course is open to all those participate in research including IRB members, research teams in the social science and health sciences as well as political authorities etc. Major topics include; history, core values and concepts of ethics for research involving humans, framework, national instruments, institutional requirements and introduction to research ethics evaluation. Teaching methods comprise; online lectures, case studies, text, videos and other resources. For assessment, participants are expected to provide correct answers in a quiz or test. The course may be completed in 3–8 h and it is free. A certificate is awarded upon completion of the course.

**JHSPH Open Courseware -Ethics of Human Subjects Research**: http://ocw.jhsph.edu/index.cfm/go/viewCourse/course/EthicsHumanSubject/coursePage/index/

This 2 credit course introduces students to the ethics of human subjects’ research. The course is divided into junks of information completed in eight weeks. The first week of course focus on ethical theory. The rest of the week’s cover substantive issues relevant to the conduct of human subject research. Major topics include; ethical theory, principles, history, contemporary issues in research ethics, balancing the benefits and harms of participating in research, ethical issues in study design, informed consent in research, Institutional Review Board. Teaching methods comprise lectures, moderated discussions, readings and case study analysis. Lecture materials, readings are available for students. Evaluation is based on participation in moderated discussions, informed consent exercise and written case analysis. The course takes 8 weeks to complete.

**Family Health International (fhi360) Research Ethics Training Curriculum:** http://www.fhi360.org/sites/all/libraries/webpages/fhi-retc2/RETCTraditional/intro.html

This course is designed to train researchers, IRB committee and members of the public who are interested in reviewing and implementing research that includes human participants. The content is subdivided by topics and subtopic. The sections are enhanced by pictures and images drawn from different countries. Major topics include; principles, development of contemporary research ethics, informed consent, responsibilities of Research Ethics Committees, responsibilities of sponsors and researchers, Community participation in the research process. Teaching methods comprise slides for online presentation, case study analysis and discussion points. There is an option for facilitator if need be. Assessment methods used include a post-test and a curriculum evaluation form. The course could take 3–8 h to complete and it is free. Certificate is awarded upon completion of this course.

**Global Health Training Centre – Research Ethics Online Training:** https://globalhealthtrainingcentre.tghn.org/elearning/research-ethics/

The course is primarily produced for the World Health Organization (WHO) internal staff; but made available for interested researchers. The content is divided into 14 topics. Major topics include; principles and guidelines, evaluating risks and benefits, issues of study design, vulnerability, engaging with the community, informed consent, bio banks and genomic research, women in biomedical research and research in public health emergencies. Variety of teaching methods is used namely, case studies, videos and resource library available and other resources. It is not easy to navigate through each module. Assessment is through an inbuilt quiz to test knowledge. The course could take 3 to 8hrs to complete and it is free. Certificate is awarded upon completion of the course.

**CITI Program – Responsible Conduct of Research (RCR**): www.citiprogram.org/

The course is intended to provide a foundation for institutions that are working to promote Responsible Conduct of Research (RCR). It is designed for undergraduates, graduates and postdoctoral researchers, administrators, IRB members and other researchers. The course is divided into 12 topics and subtopics. Major topic include; authorship, collaborative research, conflicts of interest, data management, financial responsibility, mentoring, peer review, plagiarism, research involving human subjects, research misconduct, and using animal subjects in research. Teaching methods comprise case studies, video, slide presentation. The CITI course could be used to complement an in-person training experience. Students are expected to pass each module quiz with 70% or above. The course could take 3–8 hrs. to complete and each independent learner is charged $110/course whereas USD $3500/year paid by the institution. Certificate is awarded at the end of the course.

**CITI Program-Human Subjects Research (HSR – Biomedical**): www.citiprogram.org/

This course provides an introduction to biomedical research with a focus on the protection of human subjects. It is designed for undergraduate, graduate students, postdoctoral researchers, administrators and IRB members. It is divided into 16 topics. Major topics include; history, IRB review process, informed consent, social and behavioral research (SBR) for biomedical researchers, records-based research, genetics research in human populations, risks, vulnerable populations, avoiding group harm, FDA –regulated research, privacy and protections. Teaching methods include case studies, video, slide presentation. The CITI course could be used to complement an in-person training experience. For assessment participant must pass each module quiz with 70% or above. The course could take 3–8 h to complete and each independent learner is expected to pay USD $110/course whereas institutions are charged USD $3500/year. Certificate is awarded at the end of the course.

**CITI Program – Human Subjects Research (HSR – Socio-behavioral-education)** www.citiprogram.org/

This course provides an introduction to social-behavioral-educational research with a focus on the protection of human subjects. It is designed for undergraduate, graduate students, postdoctoral researchers, administrators and IRB members. The course is divided into 13 topics. Major topics include; history, ethical principles, defining research with human subjects, the federal regulations, assessing risk, informed consent, privacy and confidentiality, research with prisoners, children and public elementary and secondary schools, international research and internet based research. Teaching methods include case studies, video, slide presentation and other resources. Students are expected to each module quiz with 70% or above. The course could take 3–8 h to complete. Each independent learner must pay USD $110/course whereas institutions pay USD $3500/year. Certificate is awarded at the end of the course.

**University of Maryland-Online Research Ethics Certificate**: http://graduate.umaryland.edu/research-ethics/

The goal of the 12 – credit, six course certificate programs is to provide individuals with an understanding of the ethical and regulatory aspects of human subjects’ research and to provide them with the skills needed to analyze ethical issues that arise in the conduct and coordination of research in domestic and international arenas. The courses offered are as follows: Introduction ton Research Ethics – 3 credits, Issues in International Research Ethics – 3 credits, Introduction to Ethical Theory – 2 credits, Institutional Review Board – 2 credits, Responsible Conduct of Research in International Affairs – 1 credit and Ethics of Globalization – 1 credit. Teaching is through online instruction and students are expected to complete, course work assignments and case analysis. Participants can complete the course in one year; but are allowed up to 3 years to complete the program. Cost charged is US$ 766 cost per credit hour for out of State learners. Certificate is awarded at the end of the course.

**University of Montana with Office of Research Integrity – Online Research Ethics Course:** https://ori.hhs.gov/education/products/montana_round1/research_ethics.html

The course is intended to provide a foundation for institutions that are working to promote responsible conduct for research. The course is divided into 6 sections and subtopic. Major topics include; ethical issues in research, interpersonal responsibility, institutional responsibility, professional responsibility, animals research and human participation in research. Teaching methods include case studies and assessment is through inbuilt quiz to test knowledge. Certificate is once participants successfully complete all the section. The course is free and duration is 3 to 8 hrs. The course has some content on introduction to research ethics; however it focuses primarily on responsible conduct of research.
